# Nondestructive Characterization of Internal Defects in Prebaked Anodes Using Open Electrical Impedance Tomography

**DOI:** 10.3390/s26196225

**Published:** 2026-09-30

**Authors:** Ziyang Huang, Xiaosong Zhou, Mingquan Qiu, Tongchao Luo, Zuhuai Wu, Rong Xiang, Qi Zhao

**Affiliations:** 1 School of Physics and Mechatronic Engineering, Guizhou Minzu University, Guiyang 550025, China; 2 Guiyang Aluminum Magnesium Design & Research Institute Co., Ltd., Guiyang 550081, China

**Keywords:** prebaked anodes, internal defects, nondestructive evaluation (NDE), open electrical impedance tomography (OEIT)

## Abstract

Prebaked anodes are critical consumables in primary aluminum electrolysis, where their internal integrity dictates cell efficiency and operational stability. Conventional defect characterization relies on destructive sampling, which inherently causes material degradation and offers poor reproducibility. Existing nondestructive evaluation (NDE) techniques are frequently limited by prohibitive costs and poor in-situ scalability. To address these challenges, this study proposes a nondestructive imaging strategy using open electrical impedance tomography (OEIT). In the DC limit employed here, the method is equivalent to open electrical resistance tomography (OERT): a constant direct current is injected, so that only the resistive contrast of the anode is exploited. A dedicated OEIT mathematical model for prebaked anodes is established, systematically detailing the signal acquisition scheme, forward problem formulation, and inverse problem regularization. By integrating conventional image reconstruction algorithms, a customized measurement procedure and a specific inversion algorithm are developed to enable the visual reconstruction of internal anomalies. The efficacy of the proposed methodology is examined through numerical simulations and benchtop experiments. Results indicate that, for the two defects examined, the approach can localize defects in the in-plane directions and approximately recover their morphology, providing a preliminary basis for further development toward online inspection.

## 1. Introduction

Prebaked anodes are functional carbonaceous electrodes fabricated from calcined carbon aggregates and coal-tar pitch binders through a high-temperature baking process, serving as essential consumable components in the Hall–Héroult aluminum electrolysis process. The internal quality of these anodes critically governs the operational efficiency and stability of reduction cells [1,2]. Internal defects compromise the electrical conductivity and mechanical integrity of the anode, potentially leading to localized overheating, equipment malfunction, and unscheduled cell outages during service. Furthermore, trace elemental constituents—notably sulfur—contained in defective anodes may evolve into hazardous gaseous species, thereby posing risks to environmental quality and occupational safety [3,4]. Consequently, the reliable evaluation of internal defects in prebaked anodes is of substantial engineering importance.

Methods for detecting internal defects in prebaked anodes fall into two principal categories: conventional destructive techniques and nondestructive evaluation (NDE) methods. The predominant conventional approaches are core drilling and manual hammering. In core drilling, a cylindrical sample is extracted using specialized tooling to evaluate the internal integrity of the carbon block. This procedure is inherently destructive, costly, and time-intensive; moreover, it typically samples only approximately 1% of the production volume, yielding limited statistical representativeness [5,6]. Manual hammering identifies defects by impacting the anode surface and interpreting the characteristics of the resulting acoustic response; this approach relies substantially on operator-dependent subjective judgment and consequently exhibits low repeatability and poor inspection consistency [7]. To overcome these constraints, extensive research efforts have been devoted to developing NDT techniques capable of efficient, non-invasive internal defect identification. The principal NDT modalities include ultrasonic testing [8], radiographic testing [9], visual testing and electrical resistivity testing [10]. Mu et al. [11] utilized the differential acoustic propagation behavior across dissimilar media to develop a customized automated hammering system integrated with machine learning for defect detection. Rodrigues et al. [12,13] employed ultrasonic methods and realized internal defect identification based on path interpolation and wavelet-based signal processing, respectively. Rørvik et al. [14] further applied X-ray imaging, thereby enabling the detection of micro-scale cracks. Nevertheless, X-ray methods involve expensive instrumentation and raise radiation safety concerns, which hinder their deployment for large-scale, in-line inspection. Hu et al. [15] proposed an autonomous crack-recognition robot integrating high-precision LiDAR and a depth camera, attaining 0.1 mm measurement accuracy in on-site validation. Nevertheless, vision-based techniques detect only surface defects and are thus incapable of identifying the internal anode flaws discussed below. Given the high electrical conductivity of prebaked anodes, Rouget et al. [16] and Kocaefe et al. [17] investigated electrically based inspection approaches. By injecting a constant current, they measured the potential differences between multiple opposing surfaces and analyzed the resulting potential distributions to infer the presence of defects. Relative to other NDE modalities, electrical methods offer rapid response and low operational cost, thus exhibiting considerable promise for practical industrial deployment. Specifically, Mishurov et al. [18] integrated electrical excitation with electromagnetic field measurement. They injected a current of 100 A and compared the measured magnetic field with that predicted by a theoretical model. A deviation exceeding 20% was interpreted as indicating a defect. This method attained a laboratory accuracy of 82%, required an inspection time of approximately 45–50 s per anode under on-site conditions, and enabled the classification of anodes into four quality grades.

Motivated by the need to overcome the above limitations of existing techniques and by the proven applicability of electrical impedance tomography (EIT) to geophysical exploration and respiratory monitoring, this paper proposes a customized OEIT-based methodology for detecting internal defects in prebaked anodes. First, a forward mathematical model is formulated to relate the electrical conductivity distribution within the anode to boundary voltage measurements; internal defects are represented as localized conductivity anomalies. Second, to suppress image artifacts induced by measurement noise and the ill-posed nature of the inverse problem, reconstruction is performed using a Gauss–Newton iterative scheme incorporating Kotre regularization. Considering the practical constraints imposed by the large dimensions of industrial anodes and the restriction of electrode access to a single surface, a dedicated OEIT electrode configuration is devised to enable non-invasive inspection. Finally, the ability of the proposed method to detect typical defects is validated using both simulated and experimental data.

The remainder of this paper is organized as follows. Section 2 introduces the theoretical foundations of EIT. Section 3 details the proposed OEIT imaging strategy for internal defect characterization. Section 4 presents the simulation and experimental validation results, and Section 5 concludes the paper.

## 2. Theoretical Foundations

### 2.1. Measurement Principle of EIT

Originating from early-20th-century geophysical resistivity imaging, EIT was later adapted to biomedical pulmonary monitoring and now encompasses industrial process tomography, materials characterization, and structural health monitoring. The application of this method to the internal quality inspection of prebaked anodes is proposed herein, in view of its cross-disciplinary versatility.

**Figure 1:**
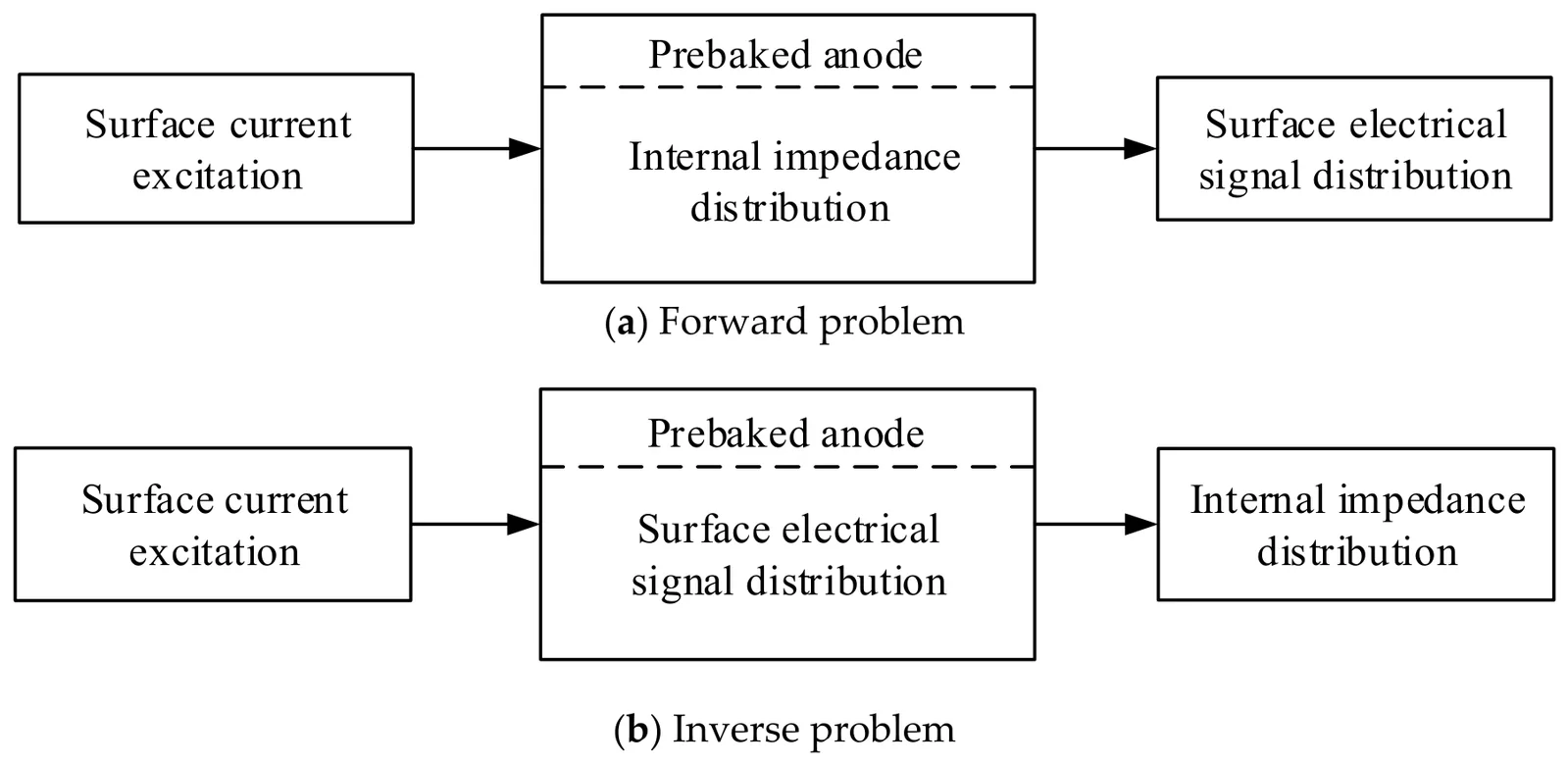
Schematic relationship between the forward and inverse EIT problems for a prebaked anode.

The physical principle underlying EIT is that the electrical resistivity of a prebaked anode serves as an indicator of its internal structural homogeneity. Upon application of an excitation current to the anode surface, both the internal current distribution and the resulting surface potential distribution are governed by the internal conductivity distribution. Consequently, measurement of surface potentials at discrete electrode locations permits the indirect inference of conductivity anomalies, thereby enabling defect identification. The relationship between the forward and inverse problems is schematically presented in Figure 1.

The prebaked anode is an electronic conductor. Its bulk resistivity is typically on the order of 50–65 μΩ×m. The relative importance of conduction and displacement currents can be assessed using $\tan\delta =\sigma /(\omega \varepsilon_{0}\varepsilon_{r})$. Even at 1 kHz, using a deliberately conservative value of $\varepsilon_{r}=1000$, tanδ is greater than $2.6\times 10^{8}$. Thus, the displacement-current term is more than eight orders of magnitude smaller than the conduction term. Under the steady DC excitation employed here, the bulk response is therefore purely resistive. The electrodes are clamped in direct contact with the carbon surface. In the implemented ideal-electrode model, each electrode is treated as an equipotential surface with perfect electrical contact; no explicit contact-impedance term is included. Interfacial capacitive transients are not part of the steady-state measurement. Consequently, the forward problem, sensitivity matrix and reconstruction are real-valued, and OEIT and OERT denote the same problem in this study.

### 2.2. Forward Problem Formulation

The electromagnetic behavior of a prebaked anode is described by Maxwell’s equations. Under the quasi-static approximation, the current field within the domain is governed by Laplace’s equation, and the mathematical model for EIT imaging of a prebaked anode can be expressed as [19]:

(1) $$\nabla \cdot \left[\sigma \left(x,y,z\right)\nabla \varphi \left(x,y,z\right)\right]=0\;\left(x,y,z\right)\in \Omega$$

where σ denotes the electrical conductivity within the anode and φ the electric potential. The electrodes are represented using the ideal-electrode approximation:

(2) $$u=U_{l}\;\mathrm{on}\;E_{l}$$

(3) $$\int_{E_{l}}\sigma \frac{\partial u}{\partial n}dS=I_{l}$$

(4) $$\sigma \frac{\partial u}{\partial n}=0\;\mathrm{on}\;\Gamma_{ins}$$

where $E_{l}$ represents the contact region between the l-th electrode and the anode, $U_{l}$ represents the equipotential value of the metal electrode, and $I_{l}$ represents the current injected into or flowing out of the electrode; for voltage measurement electrodes, $I_{l}=0$ and $\Gamma_{ins}$ represents non-electrode boundaries, including the free surface of the anode. The model assumes perfect electrical contact, but it does not include an explicit electrode–anode contact impedance.

In practical implementations, measurement noise and limited acquisition accuracy necessitate numerical approximations. The finite element method (FEM) is the most widely adopted approach. FEM furnishes a numerical solution of the forward electric field distribution and yields the sensitivity matrix, which underpins image reconstruction algorithms for the inverse problem.

(5) $$J_{pq}=\frac{\partial V_{p}}{\partial \sigma_{q}}$$

Here $V_{p}$ denotes the *p*-th boundary voltage measurement and $\sigma_{q}$ the conductivity of the *q*-th finite element. The sensitivity matrix $J_{pq}$ quantifies the influence of a small conductivity perturbation in element *q* on the *p*-th boundary voltage measurement. With the forward model thus established, the inverse problem is subsequently addressed.

### 2.3. Inverse Problem Reconstruction

The inverse problem entails reconstructing the internal conductivity distribution from the known boundary potential measurements. Based on the data formulation employed for image reconstruction, inverse methods are classified as static and dynamic modes [20]. Given that the prebaked anode constitutes a quasi-stationary system and the temporal effects of its electrochemical reactions are negligible, a static imaging reconstruction algorithm is adopted in this work.

Newton-type algorithms represent a widely employed class of optimization-based methods for static reconstruction. Their core principle lies in solving the nonlinear least-squares problem formulated from the boundary voltage residuals. Specifically, the objective is to minimize the squared $l_{2}$-norm of the residual vector between the numerically computed and experimentally measured boundary voltages [21,22,23]. Here, the $l_{2}$-norm is the Euclidean norm of the residual vector; minimizing its square is equivalent to minimizing the sum of squared voltage differences. The residual vector is defined as:

(6) $$r\left(\sigma \right)=V-F\left(\sigma \right)$$

and the corresponding least-squares objective function can be expressed as:

(7) $$f\left(\sigma \right)=\frac{1}{2}{\left\|r\left(\sigma \right)\right\|}_{2}^{2}=\frac{1}{2}r{\left(\sigma \right)}^{T}r\left(\sigma \right)$$

Here, $F\left(\sigma \right)$ denotes the boundary voltages computed from the forward model, and *V* represents the measured boundary voltages. The Gauss–Newton method transforms the nonlinear least-squares problem into a sequence of linear least-squares problems via a first-order Taylor expansion. At the current iterate $\sigma_{k}$, the forward mapping is linearized as

(8) $$F\left(\sigma_{k}+\Delta \sigma \right)\approx F\left(\sigma_{k}\right)+J_{k}\Delta \sigma$$

where $J_{k}={\left.\frac{\partial F}{\partial \sigma }\right|}_{\sigma =\sigma_{k}}$ is the Jacobian matrix. The corresponding residual approximation becomes

(9) $$r\left(\sigma_{k}+\Delta \sigma \right)\approx V-\left(F\left(\sigma_{k}\right)+J_{k}\Delta \sigma \right)=r_{k}-J_{k}\Delta \sigma$$

Substituting this approximate residual into the objective function yields:

(10) $$f\left(\sigma_{k}+\Delta \sigma \right)\approx \frac{1}{2}{\left\|r_{k}-J_{k}\Delta \sigma \right\|}_{2}^{2}$$

Minimizing this approximate function with respect to Δσ and setting the derivative to zero gives:

(11) $$\frac{\partial }{\partial \Delta \sigma }\left(\frac{1}{2}{\left\|r_{k}-J_{k}\Delta \sigma \right\|}_{2}^{2}\right)=-J_{k}^{T}\left(r_{k}-J_{k}\Delta \sigma \right)=0$$

which, upon rearranging, leads to the Gauss–Newton equation:

(12) $$J_{k}^{T}J_{k}\Delta \sigma_{k}=J_{k}^{T}r_{k}$$

The step $\Delta \sigma_{k}$ obtained from Equation (12) is then used to update the conductivity estimate:

(13) $$\sigma_{k+1}=\Delta \sigma_{k}+\sigma_{k}$$

The EIT inverse problem is severely ill-posed: a small perturbation in the measured voltage can induce a large deviation in the reconstructed conductivity. Regularization is therefore achieved by adding a penalty term to the objective function, thereby converting the ill-posed problem into a well-posed one. The regularized objective function can be written as:

(14) $$f\left(\sigma \right)=\frac{1}{2}{\left\|V-F\left(\sigma \right)\right\|}_{2}^{2}+\lambda R\left(\sigma \right)$$

where λ>0 is the regularization parameter and $R\left(\sigma \right)$ denotes the regularization functional.

Because the sensitivity of the forward problem varies by orders of magnitude between the near-electrode region and the anode interior, a uniform penalty cannot account for the differing information content of individual elements. The Kotre regularization therefore weights each element according to a power of its own sensitivity, with the exponent controlling the trade-off between the near-electrode region and the interior. Its mathematical expression is:

(15) $$R=diag{\left(diag\left(J^{T}J\right)\right)}^{P}$$

where *P* is a constant belonging to the interval $\left[0,1\right]$. When P=0, noise artifacts are distributed near the boundary; when P=0.5, noise artifacts are distributed most evenly across the domain; when P=1, noise artifacts are predominantly concentrated in the central region.

The limiting behavior follows directly from Equation (15). Let $s_{q}={\left(J^{T}J\right)}_{qq}$ denote the squared sensitivity norm for element q; then, its Kotre weight is $R_{q}=s_{q}^{-P/2}$. In general, $s_{q}$ is large for near-electrode elements and small for low-sensitivity elements in the domain interior. When P = 0, $R_{q}=1$, so the penalty is uniform and noise artifacts remain associated with the high-sensitivity boundary region. Increasing P increases $R_{q}$ preferentially for central, low-sensitivity elements; this sensitivity-weighted limiting case shifts the unresolved artifact distribution toward the domain center, whereas P = 0.5 is the heuristic intermediate case. In this work, P = 0.3 was selected empirically rather than through formal hyperparameter optimization; it provided a practical compromise between interior sensitivity and boundary artifacts in the tested datasets. A systematic parameter sweep and quantitative comparison are left as future work.

From a numerical standpoint, introducing regularization is equivalent to imposing a norm or smoothness constraint on the solution within the objective function, thereby transforming a potentially divergent or unstable iterative process into a numerically stable one.

## 3. The Proposed Methodology

**Figure 2:**
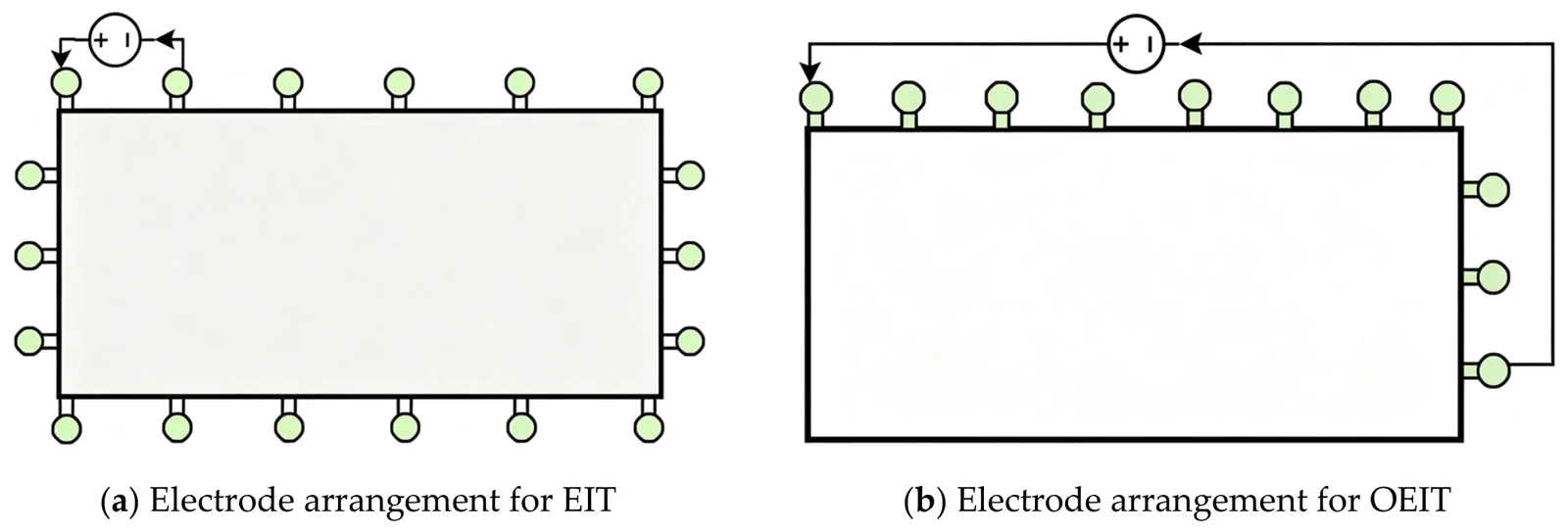
Schematic diagram of electrode arrangements for EIT and OEIT.

Conventional EIT typically requires electrodes to be arranged circumferentially around the object under test to establish a closed measurement domain. For large-scale industrial products such as prebaked anodes, however, this configuration is often constrained by limited on-site workspace, the high channel count required, and the associated cost. Open electrical impedance tomography (OEIT), by contrast, deploys electrodes on only one or several surfaces of the object, thereby forming an open measurement domain, and is consequently suited to inspection scenarios where full circumferential access is impractical. The electrode configurations for EIT and OEIT are illustrated in Figure 2.

OEIT and EIT are not distinguished by an absolute performance hierarchy; rather, they serve distinct priorities—EIT emphasizes imaging accuracy, whereas OEIT favors engineering practicality and cost-effectiveness. In this study, the selection of OEIT for identifying internal defects in prebaked anodes is motivated by two principal considerations. First, industrial field conditions impose stringent constraints on system complexity and cost, and OEIT provides an electrode configuration of reduced complexity. Second, the constrained channel count of the experimental platform available for this work renders the OEIT configuration more amenable to physical validation.

The imaging framework adopted here is that of EIT, and the term is retained in accordance with established usage. It should be noted, however, that the present implementation operates in the DC, real-valued limit, in which the reactive component of the transfer function carries no measurable defect contrast: the forward problem, sensitivity matrix, Gauss–Newton inversion and Kotre regularization are formally identical in the EIT and ERT descriptions, and the designations OEIT and OERT are therefore equivalent for the purposes of this work. The results are therefore independent of whether the OEIT or OERT designation is used.

### 3.1. Signal Acquisition Scheme

**Figure 3:**
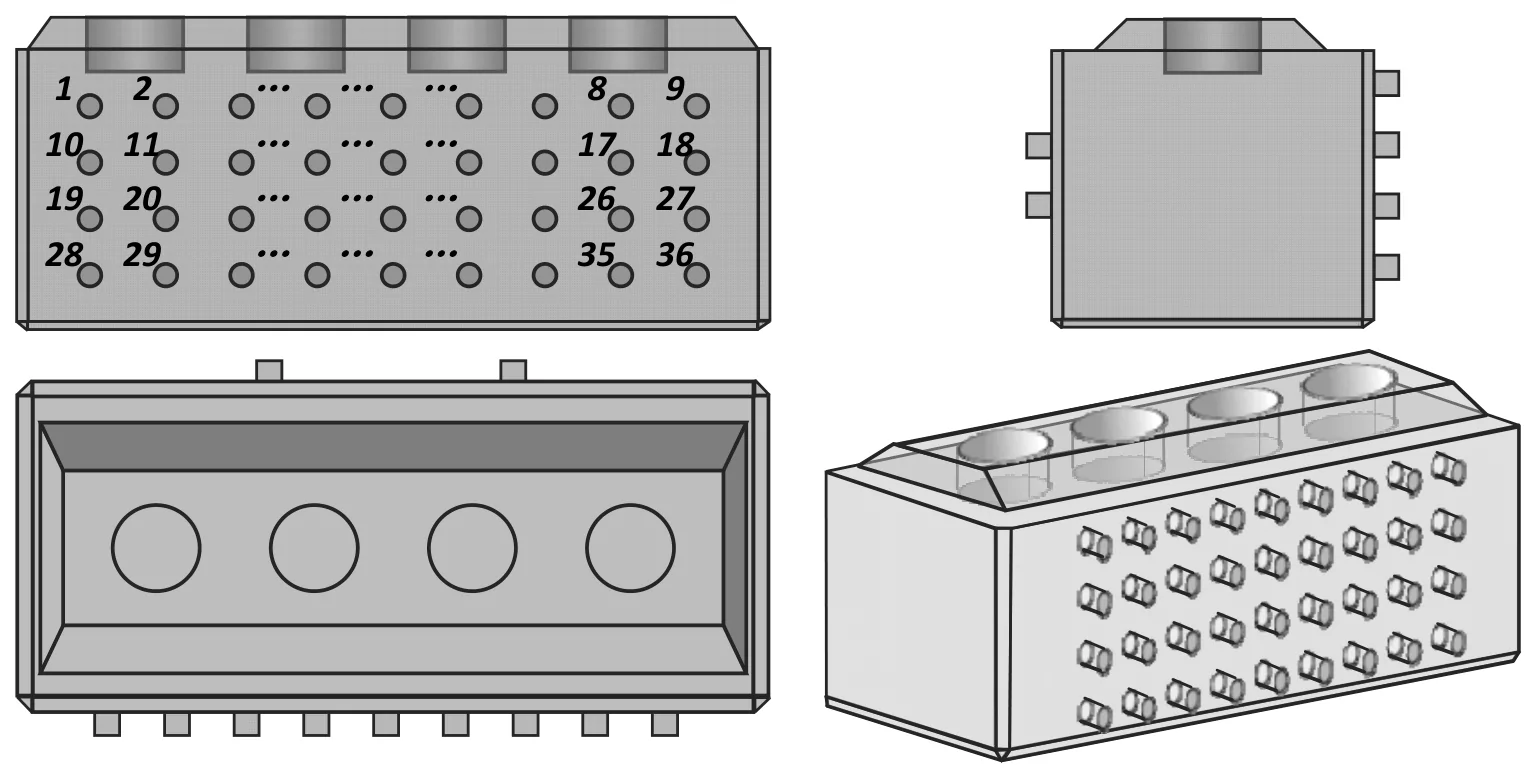
Schematic diagram of the electrode arrangement on the prebaked anode.

The electrode configuration adopted in the proposed method is illustrated in Figure 3. The prebaked anode is a rectangular parallelepiped whose length substantially exceeds its thickness. Placing electrodes on a short face would force the excitation current to propagate along the longitudinal direction; the resulting conduction path would be excessively long, causing pronounced attenuation of the measured signal. Therefore, a rectangular electrode array of 4 rows × 9 columns is deployed on one long face of the anode, confining current flow predominantly to the thickness direction. This arrangement yields a shortened conduction path and enhanced signal amplitude, thereby imparting greater measurement sensitivity to internal conductivity variations. The dense array on this surface is employed for measuring potential differences; the selection of 36 electrodes represents a trade-off between the spatial resolution of image reconstruction and the hardware cost of the electrode system. On the opposite long face, a sparse array of 2 rows × 2 columns is arranged to provide current return paths and steer the current distribution along the thickness direction, thus enabling more comprehensive interrogation of the anode interior.

In this experiment, all excitation–return electrode combinations were systematically interrogated. For each fixed return electrode, the 36 electrodes on the front face were sequentially energized as the excitation source. During each excitation, the potential differences across 27 vertically adjacent electrode pairs (rows 1-2, 2-3, and 3-4 in each column) were synchronously acquired. Specifically, current was injected through one front electrode and extracted via the fixed back return electrode, while the potential differences between the designated adjacent row pairs were measured simultaneously. Upon completion of a full scan cycle, the return electrode was switched; this process was repeated until all four return electrode configurations were completed, yielding a final dataset comprising 3888 potential difference measurements.

### 3.2. Customized OEIT Imaging Strategy for Internal Defect Visualization

The proposed internal defect visualization methodology is predicated on constructing a mapping from boundary potential measurements to the internal conductivity distribution. It comprises two principal stages: forward problem modeling and inverse problem solution. The forward problem establishes the numerical relationship between the conductivity distribution and the resulting boundary potentials, whereas the inverse problem reconstructs the internal conductivity distribution from the measured boundary potentials. The overall procedure is summarized as follows.

(1) **Data acquisition:** Following the electrode arrangement and excitation-measurement strategy detailed in Section 3.1, potential difference data are acquired from the anode surface: for each fixed back-side return electrode, the 36 front-face electrodes are sequentially energized while the potential differences across vertically adjacent row electrode pairs are synchronously recorded, thereby capturing the features of surface potential distribution.
(2) **Forward modeling:** The forward problem, derived from Maxwell’s equations under the quasi-static approximation, is governed by the Laplace equation with the boundary conditions of the ideal-electrode model. Finite-element discretization converts the resulting boundary value problem into an algebraic system; solving this system yields nodal potentials and, subsequently, the inter-electrode potential differences. Linearization of the forward model supplies the sensitivity (Jacobian) matrix, which relates boundary voltage changes to local conductivity perturbations and supports the subsequent inverse reconstruction.
(3) **Inverse conductivity reconstruction:** To minimize the residual between the measured voltages V and the forward-computed voltages U(σ), the algorithm reconstructs the relative change in resistivity via Gauss–Newton iteration incorporating Kotre regularization. At each iteration, the update step is determined from Equation (12) and the conductivity is updated according to Equation (13) until convergence of the residual is achieved. The final output is a reconstructed map of the relative change in resistivity, which is subsequently employed for defect localization and morphological characterization.

**Figure 4:**
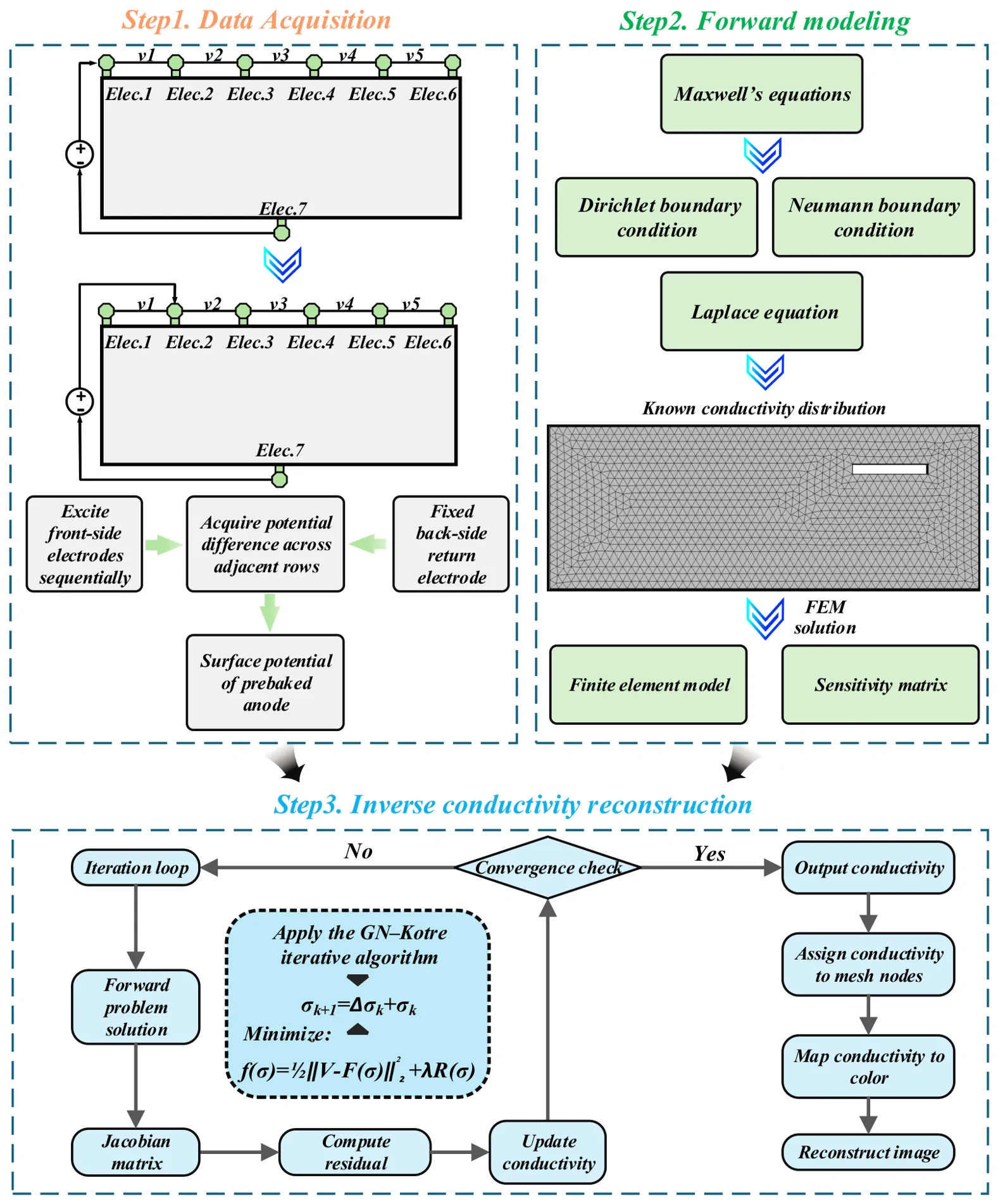
The proposed OEIT strategy for nondestructive defect characterization in prebaked anodes.

In the proposed methodology, the forward problem provides the sensitivity matrix and the requisite forward computational support for the inverse problem, whereas the inverse problem mitigates the ill-posedness through regularized iteration and reconstructs the conductivity distribution. The proposed OEIT strategy for nondestructive defect characterization in prebaked anodes is depicted in Figure 4.

## 4. Case Study

### 4.1. Simulation Analysis

#### 4.1.1. Data Generation with COMSOL

To validate the proposed method, a three-dimensional finite element model of a prebaked anode was constructed using COMSOL Multiphysics for OEIT simulations. The modeling procedure comprised the following steps. First, the anode geometry was defined according to the measured physical dimensions of 1500 mm (length) × 660 mm (thickness) × 560 mm (height) and the conductivity was set to 20,756 S/m. In accordance with the electrode configuration detailed in Section 3, 36 electrodes were arranged on one long face (front surface) and 4 electrodes on the opposite long face (back surface).

Second, a cavity defect measuring 400 mm (length) × 20 mm (thickness) × 300 mm (height) was introduced at the predetermined coordinate (x = 0 mm, y = −225 mm, z = 0 mm) according to YS/T 285-2022 [24], which defines the acceptance framework for prebaked anodes but does not specify dimensional criteria for internal defects.

Finally, the computational domain was discretized with free tetrahedral elements (299,973 elements, COMSOL Multiphysics 6.2). The excitation protocol was configured to replicate the operating parameters of a practical resistivity measurement instrument: a constant current of 100 A was injected through the designated electrode pairs, and the resulting surface potential differences were recorded. No measurement noise was added to the simulated voltages; the simulation therefore represents an idealized best case.

The reconstruction minimizes Equation (14) with the sensitivity-weighted penalty of Equation (15), using exponent P = 0.3. The regularization parameter was initialized at λ_0_ = 0.01 and reduced by a factor of 0.9 at each iteration. The iteration was terminated when the relative change in the residual fell below 1 × 10^−5^ or after a maximum of 10 iterations. The measured voltages were normalized by the excitation current.

#### 4.1.2. Simulation Validation Results

To evaluate the defect inversion accuracy of the proposed method, the normalized localization error was adopted as the metric for quantifying the overall three-dimensional positioning performance. This metric, which is widely employed in NDE for assessing reconstruction and localization precision, is fundamentally defined as the absolute positional deviation normalized by a characteristic dimension of the test object. Through such normalization, the influence of object size on the localization error was effectively eliminated, thereby enabling a fair comparison of localization accuracy across objects of varying scales.

(16) $$E_{quoted}=\frac{\left|r_{inv}-r_{true}\right|}{L_{ref}}\times 100\%$$

where $r_{inv}$ is the inversion result along the X, Y, and Z axes, $r_{ture}$ is the theoretical coordinate and $L_{ref}$ denotes the characteristic length of the anode along that same axis. In this work, *L_x_* = 1500 mm, *L_y_* = 660 mm, and *L_z_* = 560 mm, corresponding to the length, thickness and height of the prebaked anode. Equation (16) is therefore evaluated once per axis for each defect, so that each coordinate error is expressed as a fraction of the anode extent in the direction in which the error is measured.

**Figure 5:**
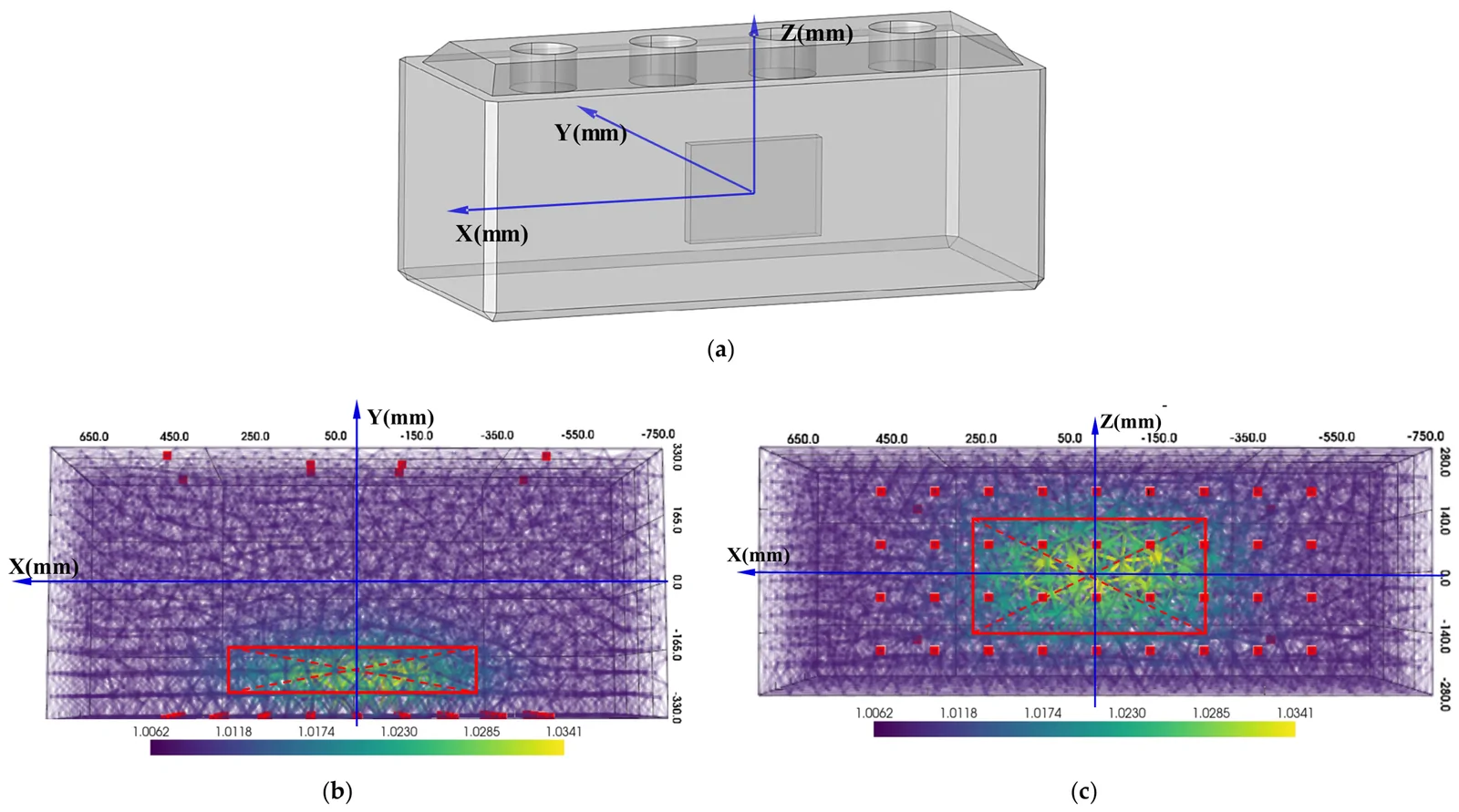
Defect imaging results for a simulated cavity defect (red box: defect region; red dots: electrode positions). (**a**) Simulated prebaked anode model containing a cavity defect. (**b**) Top view of the reconstructed conductivity distribution. (**c**) Front view of the reconstructed conductivity distribution.

The potential difference data obtained from the simulations were inverted using the reconstruction algorithm, and the defect imaging results for a simulated cavity defect are presented in Figure 5. Specifically, Figure 5a shows the simulated anode model containing a cavity defect, while Figure 5b and Figure 5c display the top and front views of the reconstructed conductivity distribution, respectively. Referenced to the Cartesian origin at the geometric center of the anode (see Figure 5a), the centroid of the reconstructed defect was located at x=10.85 mm, y=−212.33 mm and z=−12.26 mm), yielding normalized localization errors of 0.72%, 1.92% and 2.19%, relative to the nominal defect center defined in the finite element model (x=0 mm, y=−255 mm, z=0 mm). A low-conductivity region is identifiable in the reconstructed image; its contour is in good agreement with the predefined cavity defect, and its center lies within the lower portion of the anode interior.

Therefore, the capability of the proposed method for internal defect identification in prebaked anodes is corroborated by the minor localization errors along all three spatial axes and the close correspondence between the reconstructed and predefined defect morphologies, as derived from the above simulation analysis.

### 4.2. Experimental Analysis

#### 4.2.1. Experimental Setup

**Figure 6:**
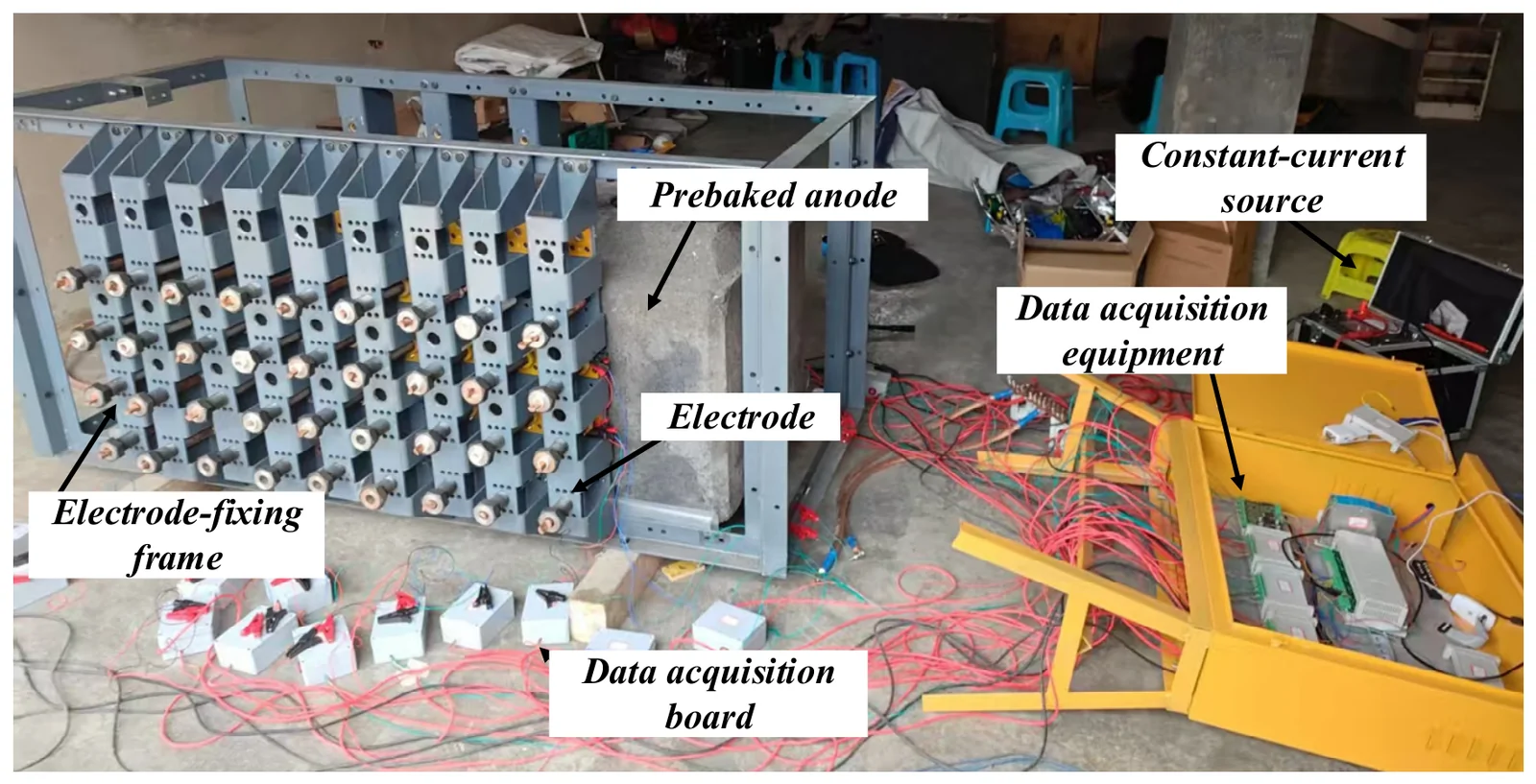
Experimental setup for electrical signal detection of prebaked anodes.

The experimental platform employed for investigating simulated defects in prebaked anodes comprises an electrode mounting fixture, the anode specimen, a constant-current source, and a data acquisition system, as depicted in Figure 6. The electrode fixture secures the measurement electrodes in reliable mechanical and electrical contact with the anode surface; an ARIT-100 electrical-resistivity measurement instrument was used to provide the regulated DC excitation and the data acquisition system recorded the resultant surface potential distribution.

#### 4.2.2. Defect Types and Locations

Before the artificial defects were introduced, baseline measurements of the intact prebaked anode were acquired using the ARIT-100 instrument. The measured bulk resistivity was $\rho_{0}=48.18\;\mu \Omega \cdot m$, corresponding to a conductivity of $\sigma_{0}=20756\;S/m$. This experimentally determined value was subsequently assigned to the intact carbon matrix in the COMSOL simulations and used as the initial conductivity estimate for inversion of the experimental data.

**Figure 7:**
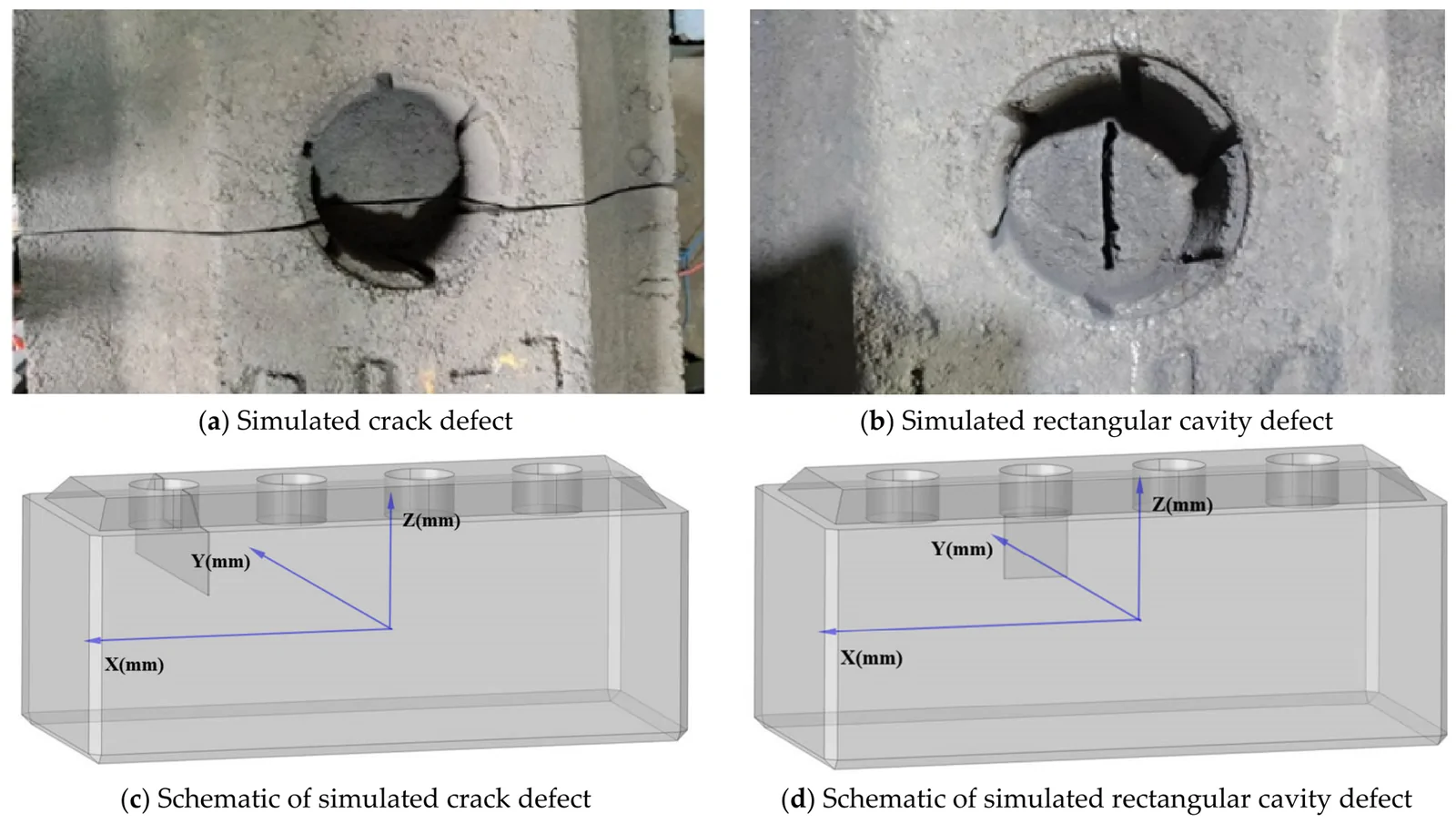
Defect types and locations in the prebaked anode.

On the upper surface of the prebaked anode, four cylindrical carbon bowls were aligned along the longitudinal direction and were sequentially numbered 1 through 4 from left to right. At bowl 1, a narrow slit measuring 3 mm in width and 200 mm in depth was introduced via wire-saw cutting perpendicular to the front face of the anode, simulating a crack-like defect. At bowl 2, a rectangular cavity was machined using an impact drill, oriented parallel to the front face to represent a loosely consolidated or detached region; the final cavity dimensions were 10 mm (thickness) × 138 mm (length) × 147 mm (depth). Both artificial defects were initiated from the center of the respective carbon bowl, and residual debris was removed after preparation, as illustrated in Figure 7.

After the crack-like defect was prepared at bowl 1, a first dataset was acquired with only this defect present, following the exhaustive excitation–measurement strategy detailed in Section 3.1. The rectangular cavity was subsequently machined at bowl 2. Since the crack could not be restored, a second dataset was acquired with both defects present simultaneously. Each dataset was inverted using the Gauss–Newton algorithm with Kotre regularization, taking the experimentally determined bulk conductivity of the anode carbon matrix as the initial estimate; the algorithm iteratively reconstructed the spatial distribution of internal conductivity variations.

#### 4.2.3. Defect Imaging Results

**Figure 8:**
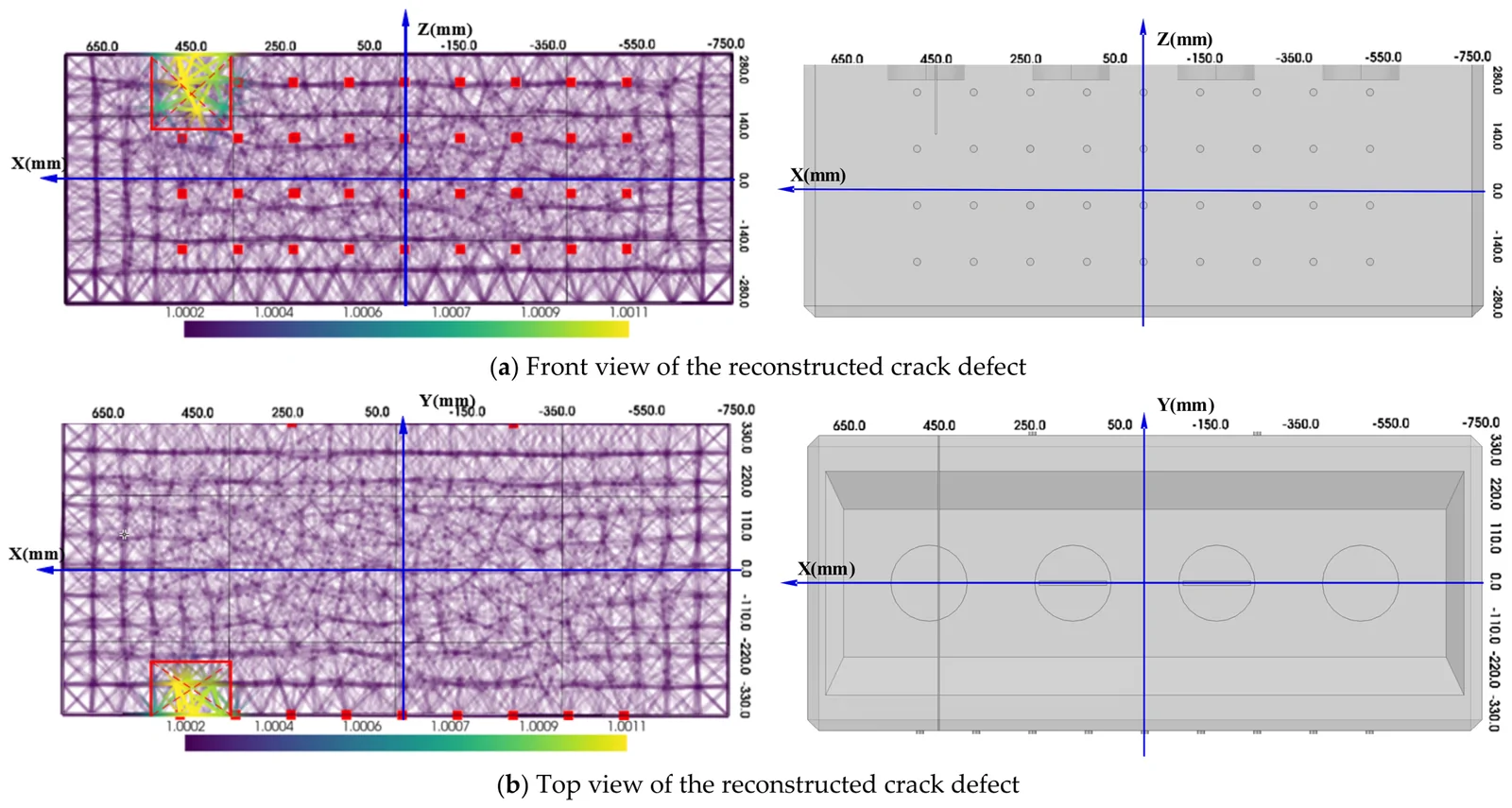
Inversion results using measured data for the crack defect (red box: defect region; red dots: electrode positions).

**Figure 9:**
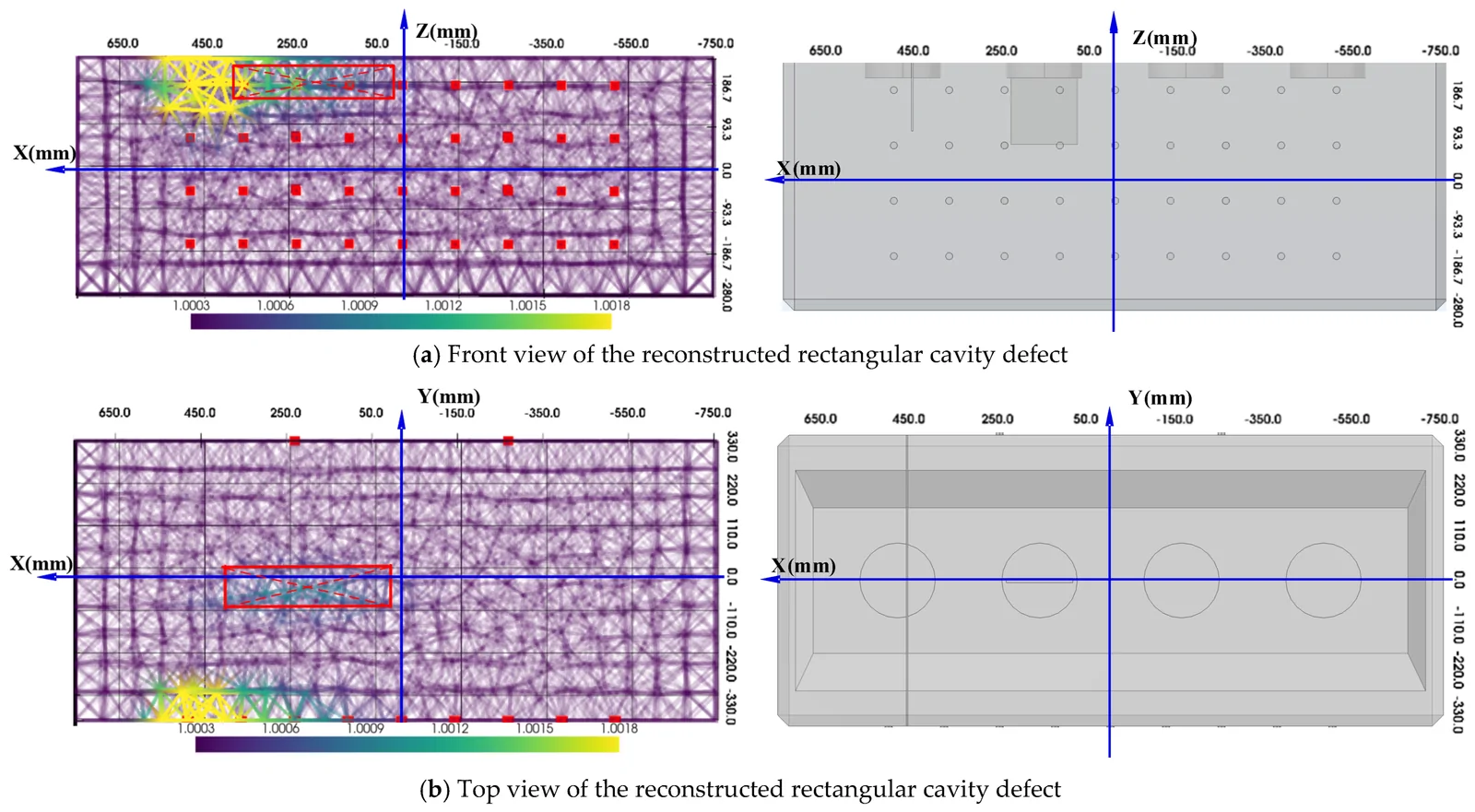
Defect imaging results with measured data for the rectangular cavity defect (red box: defect region; red dots: electrode positions).

Two imaging experiments were performed: the first with only the crack-like defect present (Figure 8), and the second after the rectangular cavity was added; as the machined crack was irreversible, both defects were present simultaneously in the second experiment (Figure 9).

The inversion results for the simulated crack-like defect are presented in Figure 8. Throughout this work, the reconstruction was presented as the relative change in resistivity with respect to the initial estimate. Higher values indicate higher resistivity relative to the background, i.e., defect locations. Within the region corresponding to carbon bowl 1, the reconstructed resistivity distribution exhibits a localized, well-defined anomaly. Adopting the geometric center of the prebaked anode as the Cartesian origin, the reconstructed defect center is located at x=465.28 mm, y=−267.45 mm and z=197.68 mm, corresponding to normalized localization errors of 0.25%, 40.52% and 1.31% with respect to the actual defect center (x=461.5 mm, y=0 mm, z=205 mm). The reconstructed image reveals an elongated, band-like high-contrast resistivity region extending along the z-direction. Its depth is broadly consistent with that of the actual crack, whereas the reconstructed width overestimates the true crack width; the feature extending along the y-axis was not resolved in the reconstruction. This imprecision arises from the crack orientation relative to the injected current. Since the current flows predominantly along the y-axis, parallel to the crack plane, the crack deflects the current only weakly, and EIT sensitivity is lowest for conductivity contrasts parallel to the current flow. The resulting weak, diffuse voltage anomaly, further smoothed by regularized Gauss–Newton reconstruction and under-resolved by electrode spacing far larger than the 3 mm crack width, accounts for the overestimated reconstructed width and the unresolved y-extent, consistent with the 40.52% normalized localization error along that axis. A more detailed discussion is provided in Section 4.3.

In addition, Figure 9 presents the inversion results for the rectangular cavity defect. The reconstructed cavity center is located at x=200.1 mm, y=−46 mm, and z=205.7 mm, yielding normalized localization errors of 2.67%, 6.97% and 0.13% relative to the actual defect position (x=160 mm, y=0 mm, z=205 mm). The reconstructed image reveals a roughly rectangular low-conductivity region that corresponds geometrically to the predefined cavity. However, the boundaries lack sharp delineation, and the reconstructed center exhibits a noticeable offset along the *x*-axis. Furthermore, because the second dataset was acquired with both defects present, the conductivity signature of the pre-existing crack defect remains discernible in Figure 9, indicating that the inversion responses arising from the two defect types are superimposed in the acquired data.

### 4.3. Discussion

The results above show that the localization accuracy of the proposed OEIT method is strongly direction-dependent. Along the in-plane axes x and z, the reconstructed centroids deviate from the true defect positions by 0.25% and 1.31% of the corresponding anode dimensions for the slit-type crack and by 2.67% and 0.13% for the rectangular cavity; in-plane localization is thus achieved at the percent level for both defects. In contrast, along the through-thickness axis y, which coincides with the direction of current injection, the normalized errors rise to 40.52% for the crack and 6.97% for the cavity.

This directional dependence follows directly from the sensitivity of the measurement configuration. From the reciprocity-based sensitivity relation, the change in a measured potential difference produced by a conductivity perturbation is:

(17) $$\Delta V_{p}=-\int_{\Omega }\delta \sigma \;\nabla u_{p}\cdot \nabla v_{p}\;d\Omega$$

where $u_{p}$ is the field due to the injected current and $v_{p}$ is the adjoint (measurement) field. For a thin insulating slit of normal $\hat{n}$, the leading contribution reduces to

(18) $$\Delta V_{p}\propto -\sigma \int_{s}\;(\hat{n}\cdot \nabla u_{p})(\cdot \nabla v_{p})\;dS$$

so that a slit whose plane contains the dominant direction of ∇u produces a vanishing first-order response. In the configuration of Section 3.1, current is injected through a front-face electrode and returned through an electrode on the opposite face, so that the interior current density is predominantly along the through-thickness (y) direction for every measurement. The machined slit lies in a plane parallel to y, with its normal in the x–z plane, so that $\hat{n}\cdot \nabla u_{p}$ is small over the entire slit surface and the boundary response is correspondingly weak. The configuration is therefore intrinsically more sensitive to cracks perpendicular to y than to cracks parallel to it. The high aspect ratio of the slit also induces a more pronounced volumetric smearing than that of the compact cavity, which further amplifies the apparent centroid shift along y.

Beyond directional sensitivity, three additional limitations of this feasibility study should be noted. First, the homogeneous background conductivity was measured on the same anode in its intact state, before any defect was introduced, and all reconstructions were referenced to this same-block healthy-state background. The homogeneous value therefore represents an effective bulk conductivity rather than that of a microscopically uniform material. Because the block-specific aggregate–pitch composition and global bulk conductivity are common to the baseline and defect measurements, same-block referencing removes block-to-block variability; nevertheless, strong local aggregate–pitch heterogeneity may still resemble a defect and generate false alarms, as the static reconstruction simply identifies spatially coherent deviations from the effective background.

Second, the reconstructions were obtained solely from Gauss–Newton inversion combined with Kotre regularization, without any quantitative comparison with Tikhonov or total-variation regularization, Landweber iteration, or data-driven reconstruction methods.

Third, the forward model adopts the ideal-electrode approximation and neglects the electrode–anode contact impedance.

These limitations indicate several directions for future work. Complementing the through-thickness current injection with in-plane excitation along the x- and z-axes would ensure that a crack parallel to one injection direction is approximately perpendicular to another, and fusing the reconstructions from multiple excitation directions would substantially improve sensitivity to arbitrarily oriented defects; increasing and optimizing the electrode density, particularly around the carbon bowls, would further improve spatial resolution. A reference-based time-difference imaging mode, validated on multiple intact production anodes and supported by repeated baseline measurements, spatially resolved background statistics, explicit detection thresholds, and quantified false-alarm rates, would address heterogeneity-induced false alarms. A rigorous algorithm benchmark should employ identical datasets and meshes, consistent hyperparameter selection, and common localization, morphology, residual, noise-robustness and computational-cost metrics, while the contact impedance should be measured and the ideal-electrode model compared with the complete electrode model. With such optimization of the excitation and reconstruction strategy for specific anode geometries, the proposed method has the potential to become a viable tool for rapid, low-cost defect screening.

## 5. Conclusions

This study proposes a nondestructive open electrical impedance tomography (OEIT) method for characterizing internal defects in prebaked anodes. Defects are treated as localized conductivity anomalies, for which a dedicated forward model was established, and the inverse problem was solved using Gauss–Newton iteration with Kotre regularization to reconstruct the internal conductivity distribution. To accommodate the large anode dimensions and single-surface access, a customized electrode configuration was developed: a 4 × 9 array on the front face for excitation and voltage measurement and a 2 × 2 array on the opposite face for current return, combined with an exhaustive excitation–measurement strategy. Both simulations and benchtop experiments demonstrate that the method localizes the two tested defects along the in-plane directions at the percent level and approximately recovers their morphology, whereas larger deviations occur through the thickness, particularly for a crack parallel to the current direction. Although the advantages in cost and non-invasiveness were not quantified here, the method provides a preliminary technical basis for further development toward online inspection of large carbon components.

## Data Availability

The original contributions presented in the study are included in the Article. Further inquiries can be directed to the corresponding author.
